# Molecular systematics of the *Reithrodontomys tenuirostris* group (Rodentia: Cricetidae) highlighting the *Reithrodontomys microdon* species complex

**DOI:** 10.1093/jmammal/gyab133

**Published:** 2021-12-11

**Authors:** Daily Martínez-Borrego, Elizabeth Arellano, Francisco X González-Cózatl, Ivan Castro-Arellano, Livia León-Paniagua, Duke S Rogers

**Affiliations:** 1 Centro de Investigación en Biodiversidad y Conservación, Universidad Autónoma del Estado de Morelos, Avenida Universidad, Chamilpa, Cuernavaca, Morelos, México; 2 Department of Biology, Texas State University, San Marcos, TX, USA; 3 Colección de Mamíferos – Museo de Zoología “Alfonso L. Herrera”, Departamento de Biología Evolutiva, Facultad de Ciencias, Universidad Nacional Autónoma de México, Ciudad Universitaria, Ciudad de México, México; 4 Department of Biology and Monte L Bean Life Science Museum, Brigham Young University, Provo, UT, USA

**Keywords:** Cricetid rodent, Cytochrome *b*, *Fgb*, harvest mice, species delimitation

## Abstract

The *Reithrodontomys tenuirostris* species group is considered “the most specialized” within the genus *Reithrodontomys* from morphological and ecological perspectives. Previous studies based on molecular data recommended changes in the taxonomy of the group. In particular, *R. microdon* has been the most taxonomically questioned, with the suggestion that it constitutes a complex of cryptic species. We analyzed the phylogenetic relationships of the *R. tenuirostris* species group using DNA sequences from the mitochondrial Cytochrome *b* gene and Intron 7 of the nuclear beta fibrinogen gene. In addition, divergence times were estimated, and possible new taxa delimited with three widely used species delimitation methods. Finally, possible connectivity routes based on shared haplotypes were tested among the *R. microdon* populations. All species were recovered as monophyletic with the exception of *R. microdon*, whose individuals were grouped into four different haplogroups, one of which included specimens of *R. bakeri*. Diversification within the *R. tenuirostris* species group began about 3 Ma, in the Pleistocene. The bGMYC and STACEY delimitation methods were congruent with each other, delimiting at the species-level each haplogroup within *R. microdon*, while the mPTP suggested a greater number of species. Moreover, none of the haplogroups showed potential connectivity routes between them, evidencing lack of gene flow. Our results suggest the existence of a higher number of species in the *R. tenuirostris* group, because we show that there are four species within what is currently recognized as *R. microdon*.

## Abstract (es)

Dentro del género *Reithrodontomys*, el grupo de especies *R. tenuirostris* es considerado “el más especializado” morfológica y ecológicamente. Estudios moleculares previos recomendaron cambios en su taxonomía, proponiendo a *R. microdon* como un complejo de especies crípticas. Se analizaron las relaciones filogenéticas del grupo de especies *R. tenuirostris* con base en información de un gen mitocondrial, Citocromo b, y uno nuclear, el intrón 7 del beta fibrinógeno. Se estimaron los tiempos de divergencia, y se delimitó a posibles nuevos taxa aplicando tres métodos comúnmente utilizados, y se evaluaron posibles rutas de conectividad con base en los diferentes haplotipos identificados en las poblaciones de *R. microdon*. Todas las especies del grupo se recuperaron como monofiléticas excepto *R. microdon*, cuyos individuos formaron cuatro haplogrupos diferentes, uno de los cuales incluyó a especímenes de *R. bakeri*. Según la datación obtenida, la diversificación del grupo *R. tenuirostris* comenzó en el Pleistoceno, hace aproximadamente 3 Ma. Los métodos de análisis de delimitación de especies bGMYC y STACEY resultaron congruentes entre sí, logrando delimitar cada haplogrupo dentro de *R. microdon* a nivel de especie, mientras que con el método de mPTP se delimitó un número mayor de especies. No se identificaron rutas de conectividad entre haplogrupos, lo que resultaría en una ausencia de flujo génico. Se sugiere la existencia de un mayor número de especies en el grupo *R. tenuirostris*, ya que hay cuatro especies incluidas en lo que actualmente se reconoce como *R. microdon*.

Establishing boundaries among species constitutes one of the main challenges for systematics, especially in taxa for which speciation processes have not resulted in obvious morphological differentiation (Goldstein and de Salle 2011). Faced with this problem, one approach has been to use multiple lines of evidence (e.g., ecology, behavior, biogeography, and genetics) to infer species boundaries (Dayrat 2005; Padial *et al.* 2010). The incorporation of molecular data in systematics studies has been particularly useful in resolving taxonomic problems in different zoological groups, especially those where multiple cryptic species have been identified (Bickford 2007; Jörger and Schrödl 2013; Struck *et al.* 2018). Within mammals, Rodentia is a clear example of a group that comprises a relatively large number of taxonomically complex and/or insufficiently studied lineages that contain cryptic species (Fabre *et al.* 2012; Burgin *et al.* 2018). One explanation is that by exhibiting relatively high evolutionary rates, rodents reflect rapid evolution (adaptive radiation), which often is associated with convergence during evolution (Hartenberger 1985; Pagel *et al.* 1991; Triant and DeWoody 2006).

Harvest mice of the genus *Reithrodontomys*Giglioli, 1874 constitute an example of cryptic species complexes. Of the 24 species in the genus, at least six have been found to be composite based on molecular data (Sullivan *et al.* 2000; Arellano *et al.* 2003, 2005; Miller and Engstrom 2008; Hardy *et al.* 2013; Statham *et al.* 2016). These studies have revealed values of intraspecific genetic divergence above 5%, which are in the same order of magnitude of those reported for distinct rodent species (Bradley and Baker 2001). Additional studies focused on clarifying both intraspecific and interspecific evolutionary relationships therefore remain necessary (Arellano *et al.* 2005; Miller and Engstrom 2008; Gardner and Carleton 2009).

The genus *Reithrodontomys* is comprised of two subgenera: *Reithrodontomys*Giglioli, 1874 and *Aporodon*Howell, 1914. Within *Aporodon*, Hooper (1952) defined the *R. mexicanus* and *R. tenuirostris* species groups, but the existence of at least two additional species groups has been suggested (Arellano *et al.* 2005). The *R. tenuirotris* species group originally included *R. tenuirostris*Merriam, 1901 (narrow-nosed harvest mouse), *R. microdon*Merriam, 1901 (small-toothed harvest mouse), *R. creper*Bangs, 1902 (Talamancan harvest mouse), and *R. rodriguezi*Goodwin, 1943 (Rodriguez’s harvest mouse), which were considered by Hooper (1952) the most specialized in the genus, both morphologically and ecologically. Later, the species *R. bakeri*Bradley, Mendez-Harclerode, Hamilton and Ceballos, 2004 (Baker’s small-toothed harvest mouse) and *R. musseri*Gardner and Carleton, 2009 (Musser’s harvest mouse) were added to the *R*. *tenuirostris* group, and *R. cherrii* (Allen, 1891—Costa Rican harvest mouse) was shown to be more related to the *R. tenuirostris* group than to *R. mexicanus* species group (Arellano *et al.* 2003, 2005), to which it originally belonged.

Within the *R. tenuirostris* group, the most taxonomically complex species has been *R. microdon*. It currently is recognized as polytypic, with three subspecies: *R. m. microdon*Merriam, 1901; *R. m. albilabris*Merriam, 1901; and *R. m. wagneri*Hooper, 1950; each allopatrically distributed in pine-oak and cloud forests of central and southern Mexico and northern Guatemala (Hooper 1952; Hall 1981). The species is considered rare and museum records are from above 2300 m; semi-arboreal habits are considered the norm (Hooper 1952; Musser and Carleton 2005; González-Ruíz *et al.* 2007). However, a mainly arboreal preference was reported recently (González-Cózatl and Arellano 2015). Hooper (1952) indicated that *R. m. microdon*, *R. m. albilabris*, and *R. m. wagneri* were reproductively isolated from each other, but with so few morphological differences that he declined to recognize them as separate species. In their analyses based on Cytochrome *b* (*Cytb*) gene sequences, Arellano *et al.* (2005) included samples of *R. m. microdon* from east of the Isthmus of Tehuantepec and *R. m. albilabris* from western Oaxaca in Mexico. They failed to recover *R. microdon* as monophyletic. Instead, mice from east of the Isthmus of Tehuantepec were found to be more closely related to *R*. *tenuirostris*, and those from western Oaxaca were sister to *R. bakeri*, from Central Mexico. As a result, Arellano *et al.* (2005) suggested that *R. m. albilabris* should be considered a species-level taxon.

Given the complex taxonomic history of the genus *Reithrodontomys*, particularly so within the *R. tenuirostris* species group, our goals here are to assess the evolutionary relationships among its members and, based on the resulting phylogenetic patterns, to identify and delimit putative cryptic species within this group, emphasizing populations of *R. microdon*. To accomplish these objectives, we developed additional taxon and geographic sampling for both the mitochondrial *Cytb* gene and the nuclear Intron 7 of the beta fibrinogen (*Fgb*) gene.

## Materials and Methods

### Sampling

**Fig. 1.**
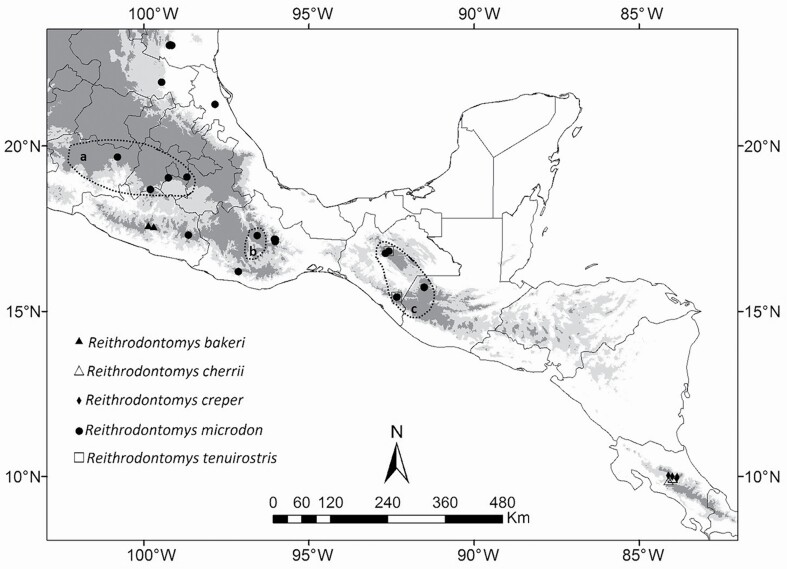
Map of Mexico and Central America showing localities for specimens of the *Reithrodontomys tenuirostris* species group analyzed in this study. Dotted dots represent the geographical distribution (proposed by Hall 1981) of the *R. microdon* subspecies [a) *R. m. wagneri*; b) *R. m. albilabris*; c) *R. m. microdon*]. Gray hues depict an elevation gradient: white <800 m; light gray 800–1700 m; and dark gray >1700 m.

Specimens used in this study were obtained from field work using methods approved in the ASM Guidelines (Sikes *et al.* 2016), by means of tissue loans from mammal collections, or from GenBank (Fig. 1; Supplementary Appendix I). For initial identifications of the wild-caught animals, we followed the morphological key developed by Hooper (1952). For the *R. tenuirostris* species group, 59 *Cytb* and 43 *Fgb* sequences were included in the molecular analyses. For both genes, the largest number of samples (40 and 32, respectively) corresponded to specimens identified a priori as *R. microdon*. The other representatives of the *R. tenuirostris* group we included were *R. bakeri*, *R. creper*, *R. cherrii*, and *R. tenuirostris*. Three species of the *R. mexicanus* group (*R. mexicanus*, *R. brevirostris*, and *R. gracilis*) and three of the subgenus *Reithrodontomys* (*R. megalotis*, *R. sumichrasti*, and *R. fulvescens*) were used as outgroups in the phylogenetic analyses. An additional 36 *Cytb* and 5 *Fgb* sequences were downloaded from GenBank (see Supplementary Appendix I).

### DNA extraction, amplification, and sequencing

Total genomic DNA was extracted from liver tissue frozen or preserved in 95% ethanol following the protocol of Fetzner (1999) or using the Qiagen DNeasy Blood & Tissue Kit extraction kit (QIAGEN Inc., Valencia, California). Polymerase chain reactions (PCRs) were undertaken to amplify the *Cytb* and *Fgb* genes. The complete *Cytb* gene (1143 bp) was amplified using the MVZ05 and MVZ14-M primers following the conditions of Smith and Patton (1993), as modified by Arellano *et al.* (2005). The combination of primers MVZ-16 (Smith and Patton 1993)–L14724 (Irwin *et al.* 1991) was used for samples with fragmented DNA. For the *Fgb* gene, 608 bp were amplified with primers B17 and Bfib (Wickliffe *et al.* 2003).

The thermal profile for the *Cytb* gene consisted of an initial denaturing of 4–5 min at 94°C, followed by 37–40 cycles of 1 min at 94°C, 1 min for the annealing at 43–45°C, and 1 min for the final extension at 72°C. For the *Fgb* gene, a 2-step touchdown was necessary. The PCR thermal profile included an initial 2 min 20 s denaturation at 94°C, followed by 45 s at 94°C, 35 s at 63°C, 1 min 30 s at 72°C (9 cycles); and 45 s at 94°C, 35 s at 53°C, 1 min 30 s at 72°C (24 cycles); and 7 min for the final extension at 72°C. Negative controls were used to ensure that there was no contamination in the products of all PCR amplifications.

The amplified PCR products of each gene were sequenced at Macrogen Inc., Seoul, Korea or at the DNA Sequencing Center at Brigham Young University. The resulting sequences were assembled and corrected by eye using the Codon Code Aligner v.8.0.2 program (CodonCode Corporation, Dedham, MA), and aligned against a reference sequence with the MUSCLE method in the UGENE v.1.32.0 program (Okonechnikov *et al.* 2012). The GenBank accession numbers for the DNA sequences generated in this study are listed in Supplementary Appendix I.

### Phylogenetic analysis

#### Cytb and Fgb data set

The model of nucleotide substitution that best fit each data set was selected with the Bayesian informative criterion (BIC), using ModelFinder (Kalyaanamoorthy *et al.* 2017). This program provides guidance about data partitioning. The models of evolution selected for the *Cytb* sequences were TIM2e + I + G, HKY + I, and TN + G for the first, second, and third codon positions, respectively. In addition, saturation of the codon positions was tested in DAMBE7 (Xia 2018). The model of evolution that best fit the *Fgb* sequences was HKY + I. These models of evolution and the DNA partition scheme (for *Cytb*) were used to estimate phylogenetic relationships in the *R. tenuirostris* species group based on the reconstructive methods of Maximum Likelihood (ML) and Bayesian Inference (BI).

Phylogenetic relationships with ML and BI were estimated in IQ-Tree (Nguyen *et al.* 2015) and MrBayes v3.2.6 (Ronquist and Huelsenbeck 2003), respectively. Both software programs were implemented within the CIPRES Science Gateway portal (Miller *et al.* 2012). In the ML analysis, branch support was calculated using 10000 Ultrafast Bootstrap replicates (UFBoot; Minh *et al.* 2013) and the GENESITE resampling strategy, which allows resample of partitions and sites within partitions (Gadagkar *et al.* 2005). Branches with UFBoot support values > 95% were considered reliable (Minh *et al* 2013). For the BI analyses, eight chains in two independent runs with 10 million Metropolis Coupled Markov Chain Monte Carlo (MCMC) generations were used. The default parameters of the model were not modified, and the trees were sampled every 1000 generations, starting the analysis with a random tree. The convergence and seasonality of each run was verified using the probability parameters in the Tracer v1.7.1 program (Rambaut *et al.* 2018), and then all trees prior the stationarity phase were discarded as burn-in. The posterior probability (pP) was obtained for individual nodes by constructing a majority-rule consensus with the trees not discarded as burn-in. Values of pP > 0.95 were considered strongly supported (Huelsenbeck and Ronquist 2001).

#### Combined data set (Cytb + Fgb)

A concatenated data set was generated (1751 bp) for the individuals in which DNA sequences for both genes could be evaluated. For these analyses, the models of evolution previously established for the *Cytb* and *Fgb* genes were used, as well as the same parameters for the phylogenetic reconstructions with ML and BI. The Wiens (1998) methodology was followed to detect inconsistencies between the resulting topologies of the phylogenetic trees with a single gene (*Cytb* or *Fgb*) and the combined data set. In this way, the branches detected with incongruities between the topologies were treated with caution (Almendra *et al.* 2014).

#### Estimation of divergence times

Divergence times between clades recovered in the *Cytb* phylogeny were estimated using BEAST2 v2.6.3 (Bouckaert *et al.* 2014). For each partition (unlinked substitution model), the same parameters and models of nucleotide substitution were established as those used in the phylogenetic analyses. The analyses were carried out using a Calibrated Yule Model prior under the assumption of an uncorrelated lognormal relaxed-clock model (Drummond *et al.* 2006), which allows variation in substitution rates between branches (Arbogast *et al.* 2002), and using the average established for mammals of 0.086 substitutions per site per million years (Steppan and Schenk 2017). In addition, for the calibration of the subgenus *Reithrodontomys* node, fossil records of the extinct species *R. moorei*, *R. wetmorei*, *R. galushai*, *R. pratincola*, and *R. rexroadensis* were integrated to construct a log-normal distribution of priors, with an offset of 1.8 Ma (M = 1.13; S = 0.3) and a probability of densities for the age of the node (HD = 95%) between 3.69 and 6.87 Ma (Dalquest 1978; Czaplewski 1987; Martin *et al.* 2002; Martin and Peláez-Campomanes 2014). The posterior distribution of the parameters, tree topology, and divergence times was determined with MCMC analysis using two runs of 10 million generations each, and trees sampled every 1000 generations. Convergence of the independent runs and the effective sample size (appropriate ESS > 200) were assessed using Tracer v1.7.1 (Rambaut *et al.* 2018). All trees prior stationary phase were discarded as burn-in, and the maximum credibility tree then was selected using TreeAnotator v2.6.2, included in BEAST2.

#### Species delimitation

We used three different methods to delimit putative new species, which allows a better interpretation of the results because congruence among the methods supports the recognition of the new taxonomic entities (Carstens *et al.* 2013). Two single-locus and one multiple-loci species delimitation methods therefore were used in this study.

Species-level lineages for the *R. tenuirostris* group were identified from the *Cytb* tree using the single-locus species delimitation methods: multi-rate Poisson Tree Processes (mPTP; Kapli *et al.* 2017) and Bayesian General Mixed Yule-Coalescent Model (bGMYC; Reid and Carstens 2012). The mPTP non-coalescent method models the branching processes, under the assumption that within species branching events will be more common, whereas among species they will be rare. This method considers the potential divergence in intraspecific diversity (Kapli *et al.* 2017) and uses the Akaike informative criterion to decide the number of resulting species according to the phylogenetic tree (Zhang *et al.* 2013). This analysis used the BI tree and the Model specifications and MCMC settings established by default in the Exelisis Lab platform (http://www.exelixis-lab.org) as input. The bGMYC coalescent method assumes that one of two events occurs at the branch points of a tree: divergence events between two species (speciation) or coalescence events between lineages within a species (Zhang *et al.* 2013). The last 100 ultrametric trees derived from the divergence time analysis were selected using LogCombiner v2.6.3 from the BEAST2 package and used as input data. The delimitation analysis was implemented in the bGMYC package (Reid and Carstens 2012) of the R library (R Core Team 2018), with the input parameters as follows: mcmc = 100000, burnin = 90000, thinning = 100, t1 = 11, t2 = 16 (based on the upper range of suggested species with mPTP, considering the outgroup), py1 = 0.5, py2 = 1.5, pc1 = 0.1, pc2 = 0.5, start = c (1.0, 0.1, 11), scale = c (20, 10, 5.00).

The Kimura 2-parameter (K2P; Kimura 1980) genetic distances for *Cytb* were estimated between the lineages suggested as new species using MEGA X (Kumar *et al.* 2018). This model of evolution allowed us to make intra- and inter-specific comparisons with genetic distance values reported in rodents (Bradley and Baker 2001; Baker and Bradley 2006).

The Species Tree and Classification Estimation, Yarely (STACEY, Jones 2017) coalescent method was used to delimit possible new species from the combined DNA sequence data (*Cytb* + *Fgb*). This multiple-loci method does not require a priori assignations of individuals to species and does not require a guide tree. Also, the number of delimited species (minimal cluster tree) can vary from one to the total number of terminals (Jones 2017). The STACEY analysis uses the birth-death-collapsed tree model as a prior and is implemented as a package within BEAST2. The SpeciesDelimitationAnalyzer program (speciesDA.jar, www.indriid.com) was used to summarize the tree posterior distribution and calculate the frequency with which each pair of taxa were assigned to the same clade. The species.tree file generated by STACEY (burnin = 1000 collapse height = 0.0001, and similarity cutoff = 1.0) was used as input data.

#### Population connectivity based on shared haplotypes

Genetic connectivity patterns were explored between the demarcated species within *R. microdon* by the bGMYC and STACEY delimitation methods (see Results section). We used the method implemented by Chan *et al.* (2011), which integrates ecological niche models (ENM) and haplotype networks to estimate putative dispersal corridors based on habitat suitability and shared haplotypes. This analysis allows making inferences about the existence of gene flow (population connectivity) or, on the other hand, the identification of possible barriers to dispersal (Chan *et al.* 2011). In addition, hypotheses about speciation processes among populations that display high genetic divergence can be corroborated.

TCS haplotype networks for *Cytb* and *Fgb* genes were constructed using PopARTv1.7 (Leigh and Bryant 2015) with 1000 permutations. This program allowed us to obtain the frequency of each haplotype, as well as the genealogical relationships among them. For the ENM, 8 Worldclim bioclimatic layers (Hijmans *et al.* 2005; Supplementary Data SD1) with a spatial resolution of ~ 1 km^2^ were used as environmental predictors. Points of occurrence for *R. microdon* were taken from SNIB-CONABIO project No. JM043 (González-Cózatl 2014), and each geographic coordinate was rectified against the known distribution (Hooper 1952; Hall 1981; González-Cózatl and Arellano 2015) to reduce georeferencing errors. A spatial thinning of occurrences was undertaken to avoid autocorrelation, establishing a minimum distance of 5 km between the localities. The model calibration areas were determined with a 70 km^2^ buffer around each occurrence record. The data processing and selection of the best fit parameters for the construction of the final model was carried out in Wallace (Kass *et al.* 2018; Supplementary Data SD1) of the R library. The final model was obtained in Maxent v3.4.0 (Phillips *et al.* 2006) with 50 bootstrap replicas and keeping the maxent logistic output (range from 0 to 1).

A data set comprised of *Fgb* haplotypes and the geographical coordinates of the site(s) where they were distributed was used as input data in the landscape connectivity analysis. This data set included the haplotypes present in *R. bakeri,* given their phylogenetic position relative to *R. m. wagneri* (see below). A friction layer was generated from the ENM obtained for *R. microdon*, where the areas of little or no probability of presence of the species depicted areas of high cost for dispersal (Chan *et al.* 2011). Least cost corridors and least cost paths then were calculated among the populations analyzed. For each comparison, the lower cost paths were classified into three categories as suggested by Chan *et al.* (2011), and the least cost corridors with higher connectivity values were interpreted as probable migration routes. This analysis was carried out with the SDMtoolbox extension (Brown *et al.* 2017) in ArcGIS v10.1 (ESRI 2011). Based on the low presence of shared *Cytb* haplotypes among the populations of the *R. microdon* species complex, we did not to use this marker in the population connectivity analysis.

## Results

### Phylogenetic analysis with Cytb

**Fig. 2.**
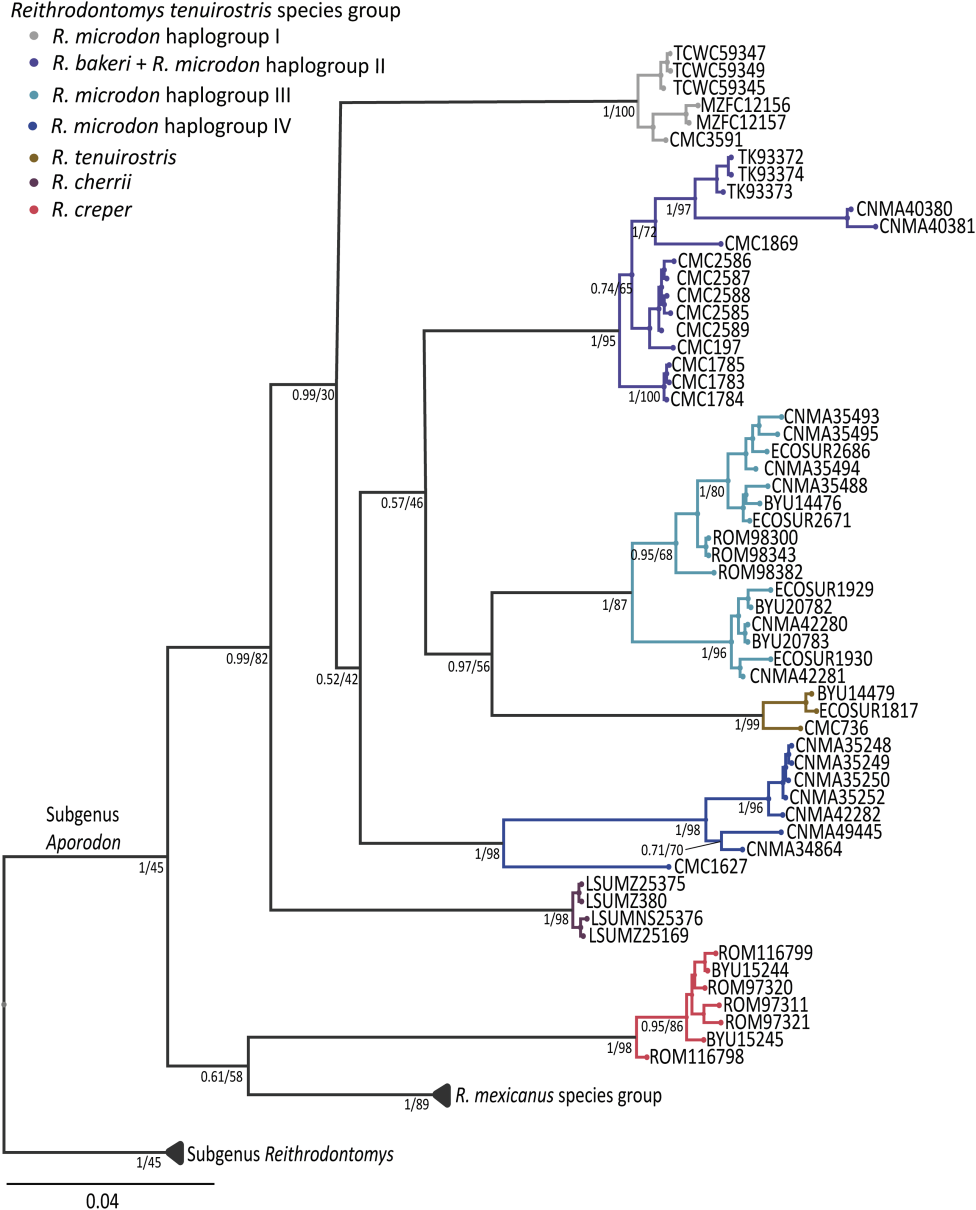
Phylogenetic relationships among species of the *Reithrodontomys tenuirostris* group using sequences data of the mitochondrial gene Cytochrome *b*. Values below branches represent nodal support for BI/ML analysis. Terminal labels are named according to mammal collection voucher numbers (see Supplementary Appendix I).

Of the 1143 nucleotides comprising the *Cytb* gene, 430 were variable; of those, 366 were parsimony-informative sites. The phylogenetic analyses with ML and BI resulted in the same tree topologies, although in general the analysis with BI showed greater nodal support (Fig. 2). In both analyses, the *R. tenuirostris* species group was recovered as a clade, with the exception of *R. creper*, which was more closely related to the *R. mexicanus* species group. Species comprising the *R. tenuirostris* group were recovered as monophyletic clades with high nodes support, with the exception of *R. microdon*.

The 40 individuals identified a priori as *R. microdon* were recovered as four haplogroups (Fig. 2; see Fig. 6 for geographic distribution of haplogroups), each strongly supported by high pP values in the BI analysis. Haplogroup I comprised samples from Tamaulipas, San Luis Potosí, and a specimen from Veracruz, Mexico. Haplogroup II was made up of individuals distributed in Michoacán, Morelos, and Estado de México, Mexico, all currently recognized as members of the subspecies *R. m*. *wagneri* together with *R. bakeri*. Haplogroup III included individuals representing the subspecies *R. m. microdon* distributed in Chiapas, Mexico and northern Guatemala. *R. tenuirostris* was recovered as the sister taxon of haplogroup III; this relationship was well supported by BI analysis (pP = 0.9), but not in the ML analysis (UFBoot = 56). Haplogroup IV included specimens classified as *R. m. albilabris* from the Oaxacan Highlands and an individual from southern Guerrero (CMC1627).

### Phylogenetic analysis with Fgb

**Fig. 3.**
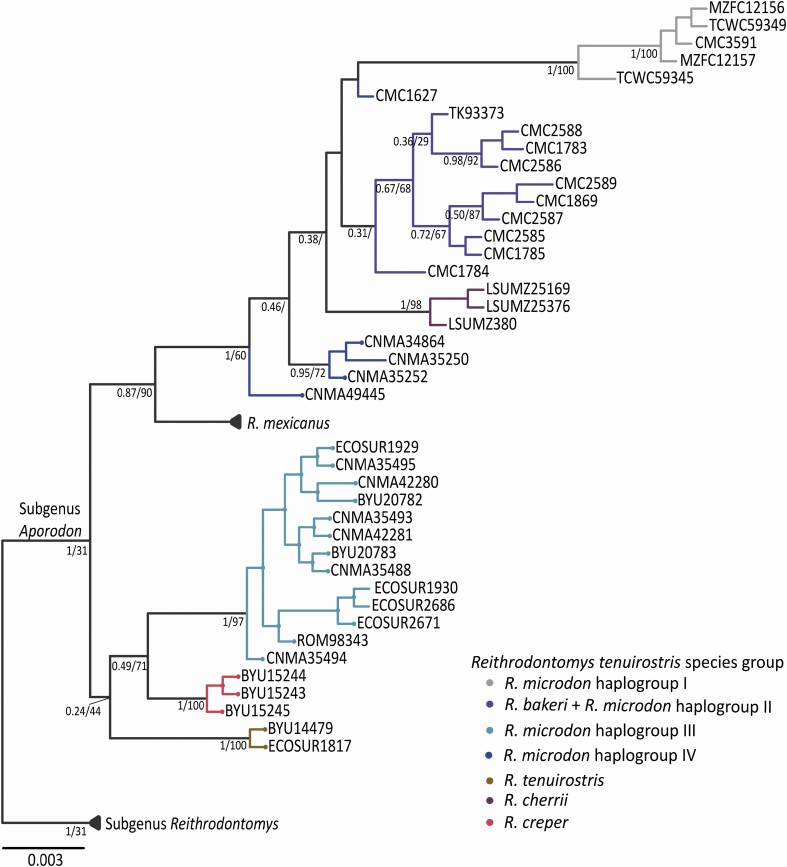
Phylogenetic relationships among species of the *Reithrodontomys tenuirostris* group using sequences data of the Intron 7 of the nuclear gene beta fibrinogen. Values below branches represent nodal support for BI/ML analysis. Terminal labels are named according to mammal collection voucher numbers (see Supplementary Appendix I).

Of the 608 nucleotides comprising the *Fgb* gene, 89 were variable, with 58 parsimony-informative sites. In addition, five regions with indels were identified: (i) at position 74, a bp deletion was inferred for haplogroup I; (ii) at position 281, a bp insertion occurred in outgroup *R. fulvescens*; (iii) at position 366, *R. creper* had a bp insertion; (iv) at position 451, there was a bp insertion for outgroups *R. megalotis* and *R. sumichrasti*; (v) at positions 563–573, a 10 bp insertion was inferred for outgroups *R. megalotis* and *R. sumichrasti*. Phylogenetic analyses with the *Fgb* nuclear intron based on ML and BI optimality criteria revealed similar tree topologies, but the BI tree was more fully resolved than the ML tree (Fig. 3). In general, both analyses showed weak nodal support, and the trees were not concordant with the phylogenetic relationships found between and within clades and haplogroups recovered in the *Cytb* tree. However, the species of the *R. tenuirostris* group were recovered in well-supported clades as well as the four haplogroups found in *R. microdon*, with the exception of haplogroup II. In addition, individuals CMC1627 and CNMA49445 did not group into haplogroup IV.

### Phylogenetic analysis of the combined data

**Fig. 4.**
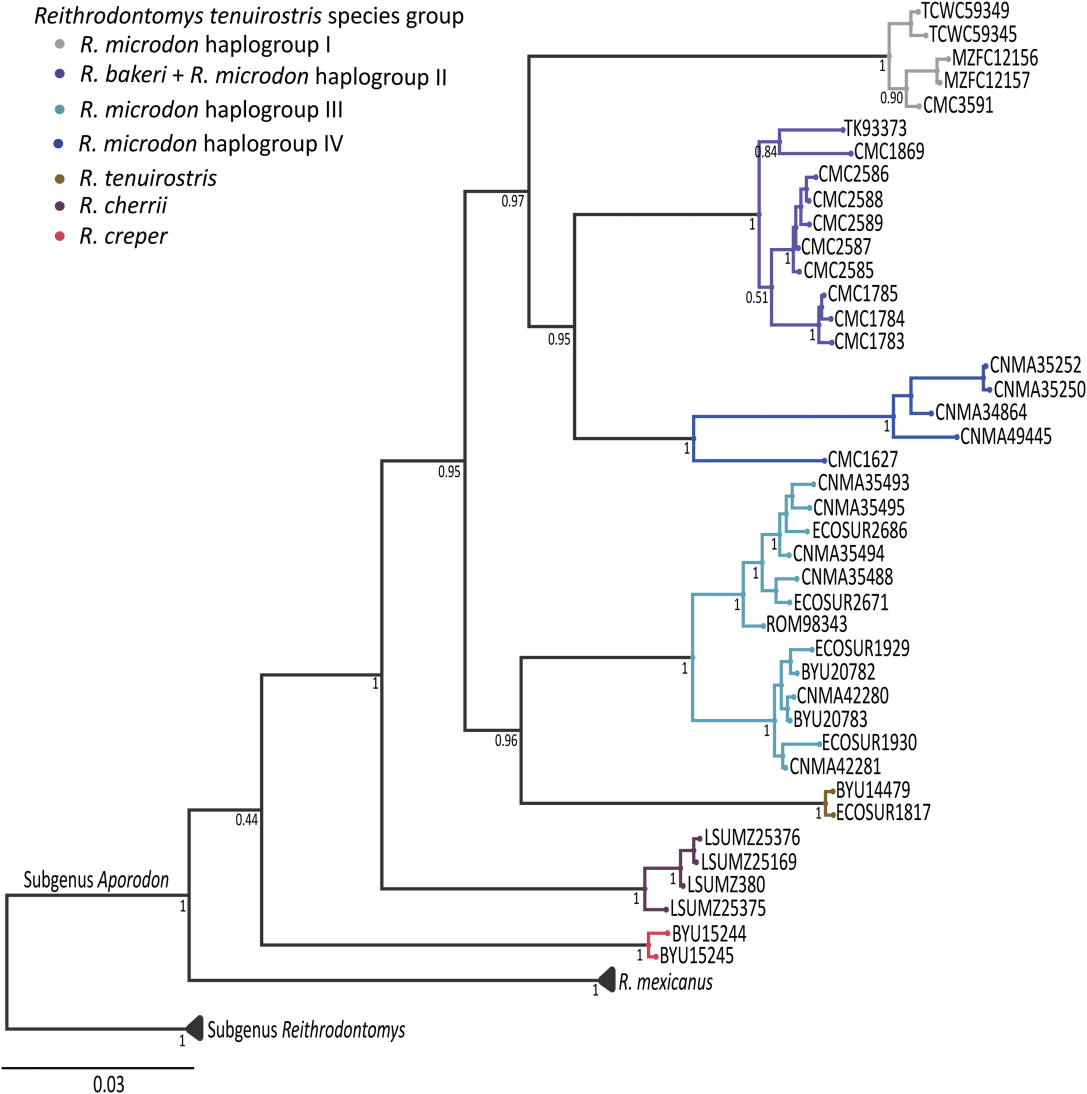
Phylogenetic relationships among species of the *Reithrodontomys tenuirostris* group using a concatenated sequences data set (Cytochrome *b* + Intron 7 of the beta fibrinogen). Values below branches represent nodal support for BI analysis. Terminal labels are named according to mammal collection voucher numbers (see Supplementary Appendix I).

Tree topologies obtained with the combined data set (1751 bp) using the BI and ML optimality criteria displayed inconsistencies between each other. The ML analysis represented essentially the same topology as the *Cytb* tree but with low UFBoot values (Supplementary Data SD2), whereas the BI tree recovered the majority of the major clades with a high nodal support (pP> = 0.95; Fig. 4). The incongruities between the BI combined and the *Cytb* tree topologies were in the positions of the haplogroup IV (*R. m. albilabris*) and *R. creper*. In the former, the samples of *R. m. albilabris* were recovered as the sister group of haplogroup II (*R. m. wagneri* + *R. bakeri*), a relationship that was well-supported (pP = 0.95). In addition, *R. creper* was recovered as the sister group of the *R. tenuirostris* species group, albeit with low nodal support (pP = 0.44).

### Estimation of divergence times

**Fig. 5.**
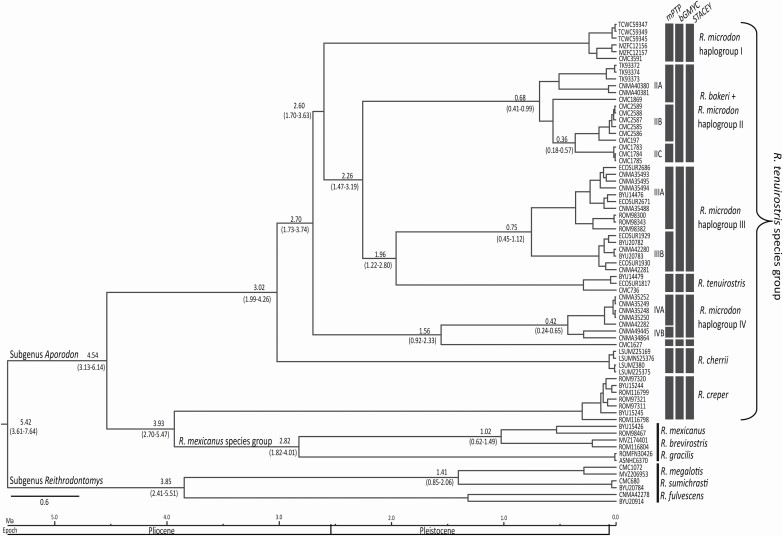
Maximum clade credibility tree obtained with BEAST2 for species of the *Reithrodontomys tenuirostris* group using Cytochrome *b* sequences data. Values above branches represent mean divergence times and below the 95% highest posterior density (HPD) intervals. Dark gray bars represent taxa delimited as species-level by the single-locus methods mPTP and bGMYC with probability values above 0.95, and the multiple-loci method STACEY.

The BEAST MCMC analysis converged on a tree topology almost identical to that obtained with the ML and BI criteria for *Cytb* (Fig. 5). The root of the most recent common ancestor (MRCA) for the genus *Reithrodontomys* had a mean age of 5.4 Ma. Within the subgenus *Aporodon* the split between the *R. tenuirostris* species group (except *R. creper*) and *R. mexicanus* was at ~ 4.5 Ma (95% HPD = 3.1–6.1). The MRCA mean age for the *R. tenuirostris* species group was estimated at 3.0 Ma (95% HPD = 1.9–4.2), when *R. cherrii* split as a different lineage. Within the four haplogroups identified for *R. microdon*, haplogroup IV diverged ~2.7 Ma (95% HPD = 1.7–3.7), whereas haplogroup I did ~2.6 Ma (95% HPD = 1.7–3.6). Haplogroup II split from haplogroup III and *R. tenuirostris* at a mean age of 2.2 Ma (95% HPD = 1.4–3.1), whereas the mean time of divergence for the latter two was estimated at 1.9 Ma (95% HPD = 1.2–2.8).

### Species delimitation

The single-locus mPTP and bGMYC delimitation methods recovered *R. creper*, *R. cherrii*, and *R. tenuirostris*, as species-level clades. Likewise, both methods inferred the presence of a species complex within *R. microdon*, although the number of delimited species (including *R. bakeri*) was not congruent across methods (Fig. 5). Specimens from Cerro Mozotal, Chiapas, Mexico were demarcated by the mPTP as a second species within haplogroup III (*R. m. microdon*), whereas the bGMYC delimited this haplogroup as a single species. The K2P genetic distance (GD-K2P; Table 1) between these putative species suggested by mPTP was 2.8%. Similarly, for haplogroup II (*R. m. wagneri* + *R. bakeri*), mPTP suggested three species-level clades, but bGMYC delimited it as a single taxon. The GD-K2P among these three lineages ranged from 1.6% to 2.4%. For haplogroup IV (*R. m. albilabris*), the mPTP and bGMYC methods delimited three (GD-K2P ranged from 1.7% to 9.2%) and two (GD-K2P 8.3%) putative species, respectively. Both methods agreed that CMC1627 represented a different species within this haplogroup. Furthermore, both delimitation methods supported haplogroup I as a species-level clade. The lineages recovered at the species-level with the multiple-loci STACEY were consistent with those delimited by the bGMYC method (Fig. 5).

**Table 1.** Matrix of Kimura 2-parameter genetic distances (%) for Cytochrome *b* gene sequence data between species of the *Reithrodontomys tenuirostris* group. Taxon labels correspond to species delimited by mPTP (A) and bGMYC (B) methods in Fig. 5.

Species delimitation											
(A) mPTP	1	2	3	4	5	6	7	8	9	10	11
1. Haplogroup I											
2. Haplogroup IIA	10.7										
3. Haplogroup IIB	10.6	1.9									
4. Haplogroup IIC	10.7	2.4	1.6								
5. Haplogroup IIIA	9.9	8.2	8.1	8.1							
6. Haplogroup IIIB	10.9	8.3	8.6	8.5	2.8						
7. Haplogroup IVA	11.8	10.2	10.5	10.9	10.6	10.0					
8. Haplogroup IVB	10.2	9.3	9.4	9.8	9.3	8.9	1.7				
9. CMC1627	11.6	7.7	9.3	8.5	8.7	8.9	9.2	7.3			
10. *R. tenuirostris*	10.5	9.1	9.6	9.7	7.7	8.5	10.3	9.5	9.4		
11. *R. cherrii*	11.6	10.6	11.5	11.4	11.2	10.5	11.5	10.7	11.0	11.1	
12. *R. creper*	12.8	13.7	13.9	14.0	12.6	13.3	13.9	13.1	13.5	13.3	12.6
(B) bGMYC	1	2	3	4	5	6	7				
1. Haplogroup I											
2. Haplogroup II	10.0										
3. Haplogroup III	9.6	6.9									
4. Haplogroup IV	10.9	9.1	8.9								
5. CMC1627	11.6	7.9	8.1	8.3							
6. *R. tenuirostris*	10.5	8.8	7.3	9.7	9.4						
7. *R. cherrii*	11.6	10.5	10.2	10.9	11.0	11.1					
8. *R. creper*	12.8	13.2	12.2	13.3	13.5	13.3	12.6				

### Population connectivity in *Reithrodontomys microdon*

**Fig. 6.**
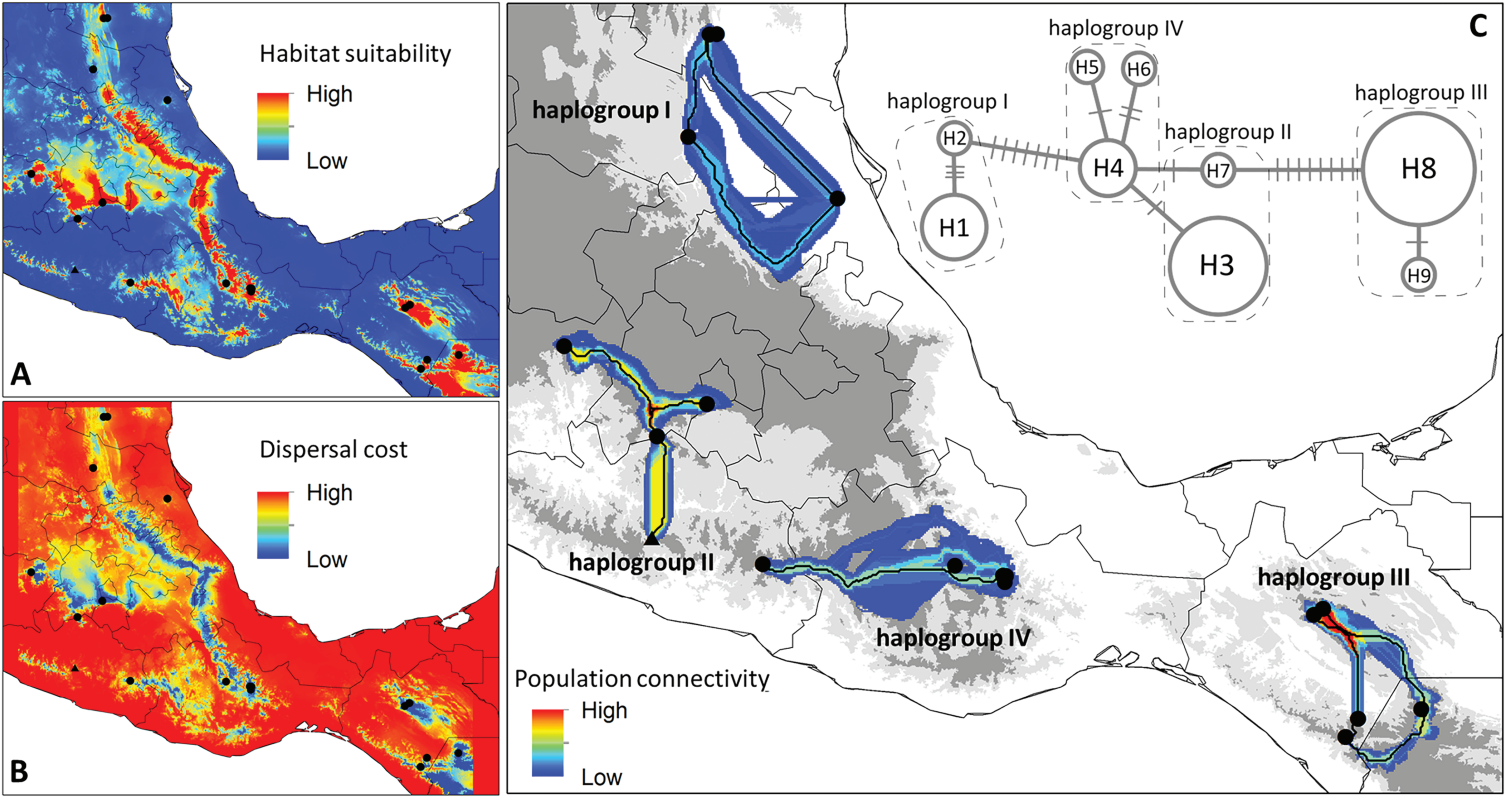
Landscape connectivity analysis based on the Intron 7 of the beta fibrinogen shared haplotypes in *Reithrodontomys microdon*. A) Ecological niche modelling of *R. microdon*; warmer colors depict high suitability areas. B) Friction layer obtained from the Ecological niche modelling; warmer colors depict areas with a high cost to dispersal. C) *Fgb* haplotype network and dispersal network with the least-cost paths; warmer colors depict paths traversed more frequently and higher population connectivity. The black circles and triangle represent the occurrence points of *R. microdon* and *R. bakeri*, respectively.

The *Cytb* haplotype network consisted of 25 haplotypes, 16 of which were exclusive to single individuals, and none shared among populations (Supplementary Data SD3). Only haplogroup III contained a haplotype shared between two localities, from northwest of San Cristóbal de las Casas, Chiapas, Mexico. The *Fgb* haplotype network consisted of nine haplotypes, five of which were exclusive to a locality, and none of which was shared among the four haplogroups (Fig. 6C).

The dispersal network among sites based on shared *Fgb* haplotypes did not show potential migration routes among the four haplogroups delimited as species-level clades by STACEY (Fig. 6C). Within haplogroup IV (*R. m. albilabris*), a possible migration route existed between populations of the Oaxacan Highlands and individual CMC1627 from Sierra Madre del Sur, but it was supported by relatively low connectivity values. Samples within each haplogroup showed cost paths crossed with a medium to low frequency, except for a low-cost path that connected the two northwestern populations of San Cristóbal de las Casas, Chiapas, Mexico. Within haplogroup II, *R. bakeri* showed a dense potential migration route with the remaining individuals of this group, in particular with those from Zacualpan, Estado de México, the geographically most proximal locality.

## Discussion

Molecular data confirm the existence of two monophyletic subgenera (*Aporodon* and *Reithrodontomys*) within *Reithrodontomys* (Howell 1914; Hooper 1952). This finding is consistent with previous molecular studies based on mitochondrial and nuclear genes (Arellano *et al.* 2003, 2005; Miller and Engstrom 2008). The mean divergence time between subgenera was the late Pliocene (5.42 Ma), followed by a relatively rapid colonization and diversification processes that favored the establishment of cryptic lineages characterized by high genetic divergence (Sullivan *et al.* 2000; Arellano *et al.* 2005; Miller and Engstrom 2008; Hardy *et al.* 2013). Within *Aporodon*, most diversification of the *R. tenuirostris* species group occurred during the Pleistocene; this finding is supported by information on environmental fluctuations and the existence of biogeographic corridors at the time (Ceballos *et al.* 2010) that favored continued expansion followed by a post-glacial isolation (Martin 1961).

The *R. tenuirostris* group currently comprises the species *R. tenuirostris*, *R. microdon*, *R. creper*, *R. bakeri*, *R. cherrii*, *R. musseri*, and *R. rodriguezi*, but the last two species were not included in this study. Our results corroborate the membership of *R. cherrii* in this species group (Arellano *et al.* 2003, 2005) but fail to support inclusion of *R. creper* because its phylogenetic relationships in the *Cytb*, *Fgb*, and combined data set trees were ambiguous. Hooper (1952) proposed that this species should be treated as part of the *R. tenuirostris* group based on morphological and ecological characteristics that make this species group the most specialized within the genus. However, he also indicated that *R. creper* was the least related to the rest of the species in this group and only shared cranial and body size measurements and coloration patterns with *R. tenuirostris* and *R. rodriguezi*, and none with *R. microdon*. Moreover, *R. creper* is morphologically distinct from species in the *R. mexicanus* group with which it overlaps in some regions, because the latter species are “much smaller; in them [N. B.: referring to *R. mexicanus* (Saussure, 1860); *R. gracilis*Allen and Chapman, 1897; *R. brevirostris*Goodwin, 1943; and *R. darienensis*Pearson, 1939] the rostrum is short and broad and the brain case is small” (Hooper 1952:176). Our results based on *Cytb* and the combined data set are partially consistent with those of Arellano *et al.* (2005), who recommended considering *R. creper* as an independent lineage within *Aporodon*. However, due to the uncertain position of this species (low values of UFBoot and Pp) among the distinct phylogenetic trees generated by the different algorithms, we were not able to support their recommendation. Phylogenetic analysis of all representatives of this species group, including other sources of evidence (morphological data), is needed to clarify the phylogenetic position of *R. creper* within the subgenus *Aporodon*.

The phylogenetic analysis with *Fgb* did not recover the same relationships among clades and haplogroups as the *Cytb* and the combined data set. Although the *R. tenuirostris* species group clade was recovered with a high nodal support, *R. mexicanus* was placed within this group, which clearly differs from the topologies generated with *Cytb* and the combined data set. Also, in the *Fgb* gene phylogeny, *R. creper* was grouped as part of the *R. tenuirostris* species group, but this was supported by low values of pP and UFBoot. These conflicting results may be due to the relatively low substitution rate that this nuclear gene presents in comparison to the mitochondrial *Cytb* gene (Wickliffe *et al.* 2003). Similar results have been reported in other rodent genera such as *Handleyomys*Voss, Gómez-Laverde and Pacheco, 2002 and *Oligoryzomys*Bangs, 1900, suggesting incomplete lineage sorting and retention of ancestral polymorphisms (Almendra *et al.* 2014; Rivera *et al.* 2018). In addition, the incongruence in topology between *Cytb* and *Fgb* trees could be explained by differences in the number of individuals and species that were used in the construction of each tree. Although at least two individuals for each clade/haplogroup obtained with *Cytb* data were represented in the *Fgb* tree, some members of the *R. mexicanus* group were missing. The absence of these species and individuals could have affected the resulting topology.

Among the four haplogroups recovered within *R. microdon*, the bGMYC and STACEY delimitated haplogroup III (*R. m. microdon*) as a species-level clade. The *Cytb* genetic distances between this haplogroup and other haplogroups or previously recognized species range from 6.9% to 9.6%. These values all were larger than the range of intraspecific divergence values reported by Bradley and Baker (2001). Although the mPTP method delimited two possible species within haplogroup III, the mean *Cytb* genetic distance (2.8%) was at the upper limit of that reported in *Reithrodontomys* for the intraspecific level (Baker and Bradley 2006). Given this relatively low genetic distance value, which coincides with a relatively short divergence time (~ 0.75 Ma), we recognize haplogroup III as a single taxon (see below). However, non-culminating speciation events (Futuyma 2013) could be occurring between the population from Cerro Mozotal, Chiapas, and the remaining populations of this haplogroup in Central Chiapas and Guatemala.

Phylogenetic relationships as ascertained with *Cytb* data recovered haplogroup IV (*R. m. albilabris*) as the sister taxon of a clade that was comprised haplogroups II, III, and *R. tenuirostris*, but this relationship was weakly supported. However, with the combined data set, haplogroup IV was closely related to haplogroup II, agreeing with Arellano *et al.* (2005), who found that *R. m. albilabris* was related to individuals of *R. bakeri* (here included in haplogroup II). The known distribution for this subspecies is restricted to the northern Oaxacan Highlands, Mexico (Hooper 1952; Hall 1981). An individual from Tejocote, Guerrero, Mexico (CMC1627) was included with this haplogroup based on its close phylogenetic relationship to specimens of *R. m. albilabris*. However, the three species delimitation methods were consistent in defining this individual as representing a distinct species-level clade, with high values of *Cytb* genetic differentiation (7.3%–11.6%) with respect to other haplogroups or species in the *R. tenuirostris* group. The divergence time between CMC1627 and the populations of *R. m. albilabris* occurred approximately 1.56 Ma (95% HPD = 0.9–2.3), which is comparable with the diversification time reported between species of various rodent genera, including *Reithrodontomys* (Schenk *et al.* 2013; Almendra *et al.* 2014; Platt *et al.* 2015).

The Oaxacan Highlands do not constitute a natural biotic unit and the affinity of the southern part of this region with the eastern part of the Sierra Madre del Sur has been previously reported (León-Paniagua and Morrone 2009). In this study, the *Fgb* dispersal network revealed a potential migration route between CMC1627 (Sierra Madre del Sur) and individuals from Cerro Zempoaltepetl (Oaxacan Highlands) by sharing the H4 haplotype, although this path showed relatively low connectivity values. The accumulated mutations in mitochondrial DNA (over 25 mutational steps with *Cytb*; Supplementary Data SD3) during the time that these populations have been geographically isolated, along with the results of species delimitation methods and the *Cytb* genetic distances, all constitute sufficient DNA evidence to suggest that CMC1627 represents an undescribed species within the *R. tenuirostris* group. However, studies including more individuals would be necessary to confirm this conclusion.

Within haplogroup IV, the mPTP method delimited the specimens CNMA49445 and CNMA34864 as a distinct species distributed in Ixtepeji and Cerro Zempoaltepec, Oaxaca, Mexico, respectively. The *Cytb* genetic distances between these two individuals (haplogroup IVB) and haplogroup IVA was 1.7%, a value lower than the range reported for rodents recognized sister species (Bradley and Baker 2001; Baker and Bradley 2006). In accordance with the results of the bGMYC and STACEY species delimitation methods and the relatively recent splits between these two groups (~0.42 Ma), we consider them as a single taxonomic group at the species level, which differs genetically from the individual CMC1627 and from the two subspecies currently recognized for *R. microdon*.

*Reithrodontomys bakeri* was described based on genetic distances of the *Cytb* gene and morphological measurements, which differentiated it from *R. microdon.* However, Bradley *et al.* (2004) compared their new species to *R. m. albilabris* and were not able to compare to the geographically most proximal subspecies of *R. microdon*: *R. m. wagneri*. In our study, we included 10 specimens of *R. m. wagneri*, distributed in the Mexican states of Michoacán, Estado de México, and Morelos. The results with *Cytb*, *Fgb*, and the combined data set showed a close relationship between these populations and *R. bakeri*, with generally high nodal support. In addition, the connectivity values of the *Fgb* dispersal network were relatively high, evidencing a potential migration route between the *R. bakeri* population from Filo de Caballo, Guerrero, and that of Zacualpan, Estado de México (*R. m. wagneri*), separated by approximately 120 km. This analysis suggests, at least for the nuclear data, the probable existence of gene flow between these populations. It could be possible that certain geographical and demographic characteristics may have favored this gene flow in the past. Although populations of *R. m. wagneri* and *R. bakeri* did not share any *Cytb* haplotypes, we assume that the relatively high rate of nucleotide substitution exhibited by mitochondrial genes compared to nuclear genes accounts for this observation (Saccone *et al.* 1999; Palumbi *et al.* 2001). Finally, *R. bakeri* and individuals representing *R. m. wagneri* were considered the same species both by the single-locus bGMYC and by the multiple-loci STACEY, with *P* > 0.95. The assumption that these populations were connected in the past also can be corroborated with *Cytb* data. The divergence mean time estimation between these populations was 0.68 Ma, which is consistent with the low degree of *Cytb* genetic differentiation found between *R. bakeri* (haplogroup IIA) and the other two groupings proposed as candidate species by the mPTP (haplogroup IIB-1.9% and haplogroup IIC-2.4%).

Haplogroup I was represented by individuals identified a priori as *R. mexicanus* given their geographic distribution north of Sierra Madre Oriental and Sierra de Otontepec in Mexico (Hooper 1952). After an exploratory analysis with the *Cytb* gene, these individuals were assigned to a part of the *R. tenuirostris* species group, and due to their morphological similarity as “*R. microdon* like.” This haplogroup was not genetically similar to any of the other haplogroups, and all the species delimitation methods recovered it as a distinct putative species. *R. mexicanus* and *R. microdon* share great similarities in cranial characteristics and pelage coloration (Hooper 1952), and they occur in sympatry in some regions in southern Mexico (Hall 1981). However, the geographical distribution of haplogroup I individuals is close to that known for *R. mexicanus* in that region, and this could explain their previous identifications as *R. mexicanus*. Otherwise, only three specimens of *R. mexicanus* have been reported from Tamaulipas, Mexico (Hooper 1952; Jones and Anderson 1958), and those records constituted the northernmost distribution for *Aporodon* at that time. The altitudinal records of those specimens are near or well below the known elevational range of *R. mexicanus* (~1,000 to 3,800 m; Martínez-Borrego *et al.* 2020) but are similar to those registered for haplogroup I specimens analyzed in this study (see Supplementary Appendix I). Given the geographical distribution and morphological similarity between the haplogroup I specimens and *R. mexicanus*, we suggest that individuals from Tamaulipas reported by Hooper (1952) and Jones and Anderson (1958) should be reclassified as part of this new candidate species.

The Sierra Madre Oriental is recognized as one of the oldest and most complex geological regions in Mexico (Ferrusquía-Villafranca 1993; Marshall and Liebherr 2000) and has been associated with cryptic speciation events in rodents (e.g., Sullivan *et al.* 2000; Arellano *et al.* 2005, Ávila-Valle et al. 2012; Almendra *et al.* 2014). The divergence times analysis estimated that the separation of haplogroup I occurred about 2.6 Ma; this group therefore has been genetically isolated from other species in the *R. tenuirostris* group for a long time. Perhaps as a consequence of similar habitats, such as the cloud forests (Gual-Díaz and Rendón-Correa 2014), members of this haplogroup maintained morphological similarities common to almost all species of *Aporodon* (Hooper 1952). Consequently, this has led to underestimating the number of species that exist in this subgenus.

Hooper (1952) noted the reproductive isolation that exists among the three subspecies of *R. microdon*, which has been corroborated in this study with the absence of possible connectivity routes among them. However, assuming little morphological differentiation, Hooper (1952) maintained them in the same species while recognizing three allopatrically distributed subspecies (Fig. 1). The subspecies *R. m. albilabris* and *R. m. microdon* are distributed in the Oaxacan Highlands and the mountains of Chiapas and northern Guatemala, respectively. Between these two regions, the Isthmus of Tehuantepec constitutes an effective geographic barrier which has presumably acted to gene flow, and thus favoring speciation events (Sullivan *et al* 2000; León-Paniagua *et al.* 2007; Esteva *et al.* 2010; Hardy *et al.* 2013), in which the high genetic divergence between populations on either side of the Isthmus has resulted in hypotheses about the occurrence of a vicariance event that affected the small mammal fauna of this Mexican region (e.g., Rogers *et al.* 2007). The distribution of *R. m. wagneri* is restricted to the Neovolcanic Belt. This may be the result of suitable habitat contractions that occurred during the Pleistocene, where these mountainous regions could have served as a refuge (Ceballos and Rodríguez 1993). In this way, they were isolated and differentiated at a specific level, a trend also reported in other genera of rodents such as *Peromyscus*Gloger, 1841 and *Microtus*Schrank, 1798 (Ceballos *et al.* 2010).

The high precision of methods for delimiting species, as well as knowledge of the assumptions made during the process (Rannala 2015), gives them advantages over empirical ways of setting limits such as using a cut-off or arbitrarily determined distance thresholds (Fujisawa and Barraclough 2013; Alström *et al.* 2021). Yet, these methods often are computationally demanding and/or their performance is potentially affected by factors that cause an under- or overestimation of the lineages to be delimited, such as variation in population sizes, number of species, ongoing gene flow, accuracy of input trees, and rate of molecular change, among others (Rannala 2015; Luo *et al.* 2018). Using alternative delimitation methods in search of congruence therefore can lead to more realistic hypotheses on species boundaries (Muñoz-Tobar and Caterino 2020). The taxonomic proposal of our study is mainly based on the results generated with three widely used delimitation methods (PTP, GMYC, and STACEY—Alström *et al.* 2021), two of which were completely congruent with each other. If we assume that speciation usually is a gradual process, then the incongruence presented by mPTP could be explained by the statistical power of each method to detect independent lineages (Carstens *et al.* 2013). Likewise, one of the main limitations of the three employed methods is the presence of gene flow (Luo *et al.* 2018). However, given that all clades representing putative new species have allopatric distributions, it is unlikely that there is ongoing gene flow between them and, as a result, the performance of these species delimitation methods would not have been affected.

Based on our results and those of Arellano *et al*. (2003, 2005), we propose that there is sufficient evidence to recognize each of the *R. microdon* subspecies as a valid species, as follows:

*Reithrodontomys microdon* Merriam, 1901

Type locality. “Todos Santos, Guatemala (altitude 10,000 ft.)”.

Synonym. *Reithrodontomys microdon microdon*Merriam, 1901:548. Type locality: see above.

Distribution. Highlands of Guatemala (Howell 1914), and the extreme southern portion of Mexico (Hooper 1952). This species is distributed from the highlands of central and southern Chiapas in Mexico to the southwestern region of Guatemala.

Remarks. For additional information on type material, cranial measurements, morphological characteristics, habitat, and comparisons with other species of the subgenus *Aporodon,* see Hooper (1952:170).

*Reithrodontomys albilabris* Merriam, 1901

Type locality. *“*Cerro San Felipe, Oaxaca, Mexico (altitude 10,000 ft.)”.

Synonym. *Reithrodontomys microdon albilabris*Merriam, 1901:549. Type locality: see above.

Distribution. Oaxacan Highlands. Known range (in addition to type locality) included Cerro Zempoaltepec, Vista Hermosa, Llano de las Flores, Cerro Pelón (Hall 1981), and Ixtepeji (this study), Mexico.

Remarks. *Reithrodontomys albilabris* comprises the specimens representing haplogroup IV, with the exception of the individual CMC1627. Information on type material and morphological characteristics is available in Merriam (1901) and Howell (1914).

*Reithrodontomys wagneri* Hooper, 1950

Type locality. “México, Michoacán, about 10 miles northwest of Ciudad Hidalgo, western 

flanks of Cerro San Andrés, 9400 feet elevation”.

Synonym. *Reithrodontomys microdon wagneri* (Hooper 1950:169). Type locality: see above.

Distribution. The Neovolcanic Belt in Mexico, with records in Michoacán, Ciudad de México, Estado de México, Morelos and Guerrero, Mexico (Hooper 1952; Hall 1981; González-Cózatl and Arellano 2015).

Remarks. We recommend inclusion within this species of the two populations from Guerrero, Mexico, recognized as *Reithrodontomys bakeri*, which according to the Principle of Priority (Article 23; ICZN 1999) should be renamed as *R. wagneri*. Also, we suggest the recognition of two subspecies within *R. wagneri*: *R. w. wagneri* and *R. w. bakeri*. For additional information on type material, cranial measurements, morphological and habitat characteristics, see Hooper (1952: 170).

Finally, haplogroup I should be considered as a candidate for a new species, with a known geographic distribution in the north of Sierra Madre Oriental (Tamaulipas and San Luis Potosí, Mexico) and Sierra de Otontepec (Veracruz, Mexico). This new species candidate represents the northernmost distribution known within the *R. tenuirostris* species group, increasing its distribution range to the mountainous regions of northeastern Mexico. Additional information sources (together with the already existing genetic information) such as morphological and ecological, among others, still are necessary to undertake the formal description of this candidate new species.

## Supplementary Material

gyab133_suppl_Supplementary_Data_1

gyab133_suppl_Supplementary_Data_2

gyab133_suppl_Supplementary_Data_3

gyab133_suppl_Supplementary_Appendix_I

## Supplementary Data

Supplementary Data SD1.—Worldclim Bioclimatic layer used to obtain the ecological niche model of *Reithrodontomys microdon* (Rodentia: Cricetidae) and the parameters of 50 ecological niche models obtained in Wallace. The best fit model parameters used to obtain the final ecological niche model are highlighted in red.

Supplementary Data SD2.—Phylogenetic relationships among species of the *Reithrodontomys tenuirostris* group (Rodentia: Cricetidae) using a concatenated sequences data set (Cytochrome *b* + Intron 7 of the beta fibrinogen) and the reconstructive method of Maximum Likelihood (ML). Values above branches represent nodal support for ML analysis. Terminal labels are named according to mammal collection voucher numbers (see Supplementary Appendix I).

Supplementary Data SD3.—TCS network for Cytochrome *b* haplotypes of *Reithrodontomys microdon* (Rodentia: Cricetidae). Haplogroup II included *R. bakeri* haplotypes. Numbers in parentheses represent mutational steps between haplotypes, and the filled gray circles are theoretical consensus sequences (unsampled or extinct).
