# Supplementary material for: Molecular systematics of the Reithrodontomys tenuirostris group (Rodentia: Cricetidae) highlighting the Reithrodontomys microdon species complex
Source: J Mammal. 2021 Dec 11;103(1):29–44. doi: 10.1093/jmammal/gyab133 (PMC8789765; doi:10.1093/jmammal/gyab133)
Supplement: gyab133_suppl_Supplementary_Data_1 [file gyab133_suppl_supplementary_data_1.docx]

Supplementary Data SD1

Worldclim Bioclimatic layer (Hijmans et al. 2005) used to obtain the ecological niche model of *Reithrodontomys microdon* (Rodentia: Cricetidae).

| Bioclimatic layer | |
| --- | --- |
| Bio2 | Mean Diurnal Range (Mean of monthly (max temp - min temp) |
| Bio6 | Min Temperature of Coldest Month |
| Bio9 | Mean Temperature of Driest Quarter |
| Bio11 | Mean Temperature of Coldest Quarter |
| Bio12 | Annual Precipitation |
| Bio14 | Precipitation of Driest Month |
| Bio16 | Precipitation of Wettest Quarter |
| Bio18 | Precipitation of Warmest Quarter |

Parameters of 50 ecological niche models, obtained in Wallace (Kass et al. 2018) for *Reithrodontomys microdon* (Rodentia: Cricetidae). The best fit model parameters used to obtain the final ecological niche model are highlighted in red. L=Linear; Q=Quadratic; H=Hinge; P=Product. AUC=Area Under Curve. MTP=Minimum training presence. AIC=Akaike Informative Criterion. RM=Regularization multiplier.

| **Features** | **RM** | **Train AUC** | **Avg test AUC** | **Avg diff AUC** | **Avg test or MTP** | **Avg test or 10pct** | **AICc** | **delta AICc** | **wAIC** |
| --- | --- | --- | --- | --- | --- | --- | --- | --- | --- |
| H | 2.5 | 0.91 | 0.89 | 0.02 | 0.01 | 0.12 | 1353.08 | 0.00 | 0.80 |
| LQHP | 3 | 0.91 | 0.89 | 0.02 | 0.01 | 0.13 | 1357.60 | 4.52 | 0.08 |
| H | 3 | 0.90 | 0.89 | 0.02 | 0.01 | 0.12 | 1359.93 | 6.85 | 0.03 |
| LQHP | 2.5 | 0.91 | 0.90 | 0.02 | 0.01 | 0.15 | 1360.18 | 7.10 | 0.02 |
| H | 2 | 0.91 | 0.89 | 0.02 | 0.01 | 0.12 | 1361.44 | 8.36 | 0.01 |
| H | 4 | 0.90 | 0.89 | 0.02 | 0.01 | 0.13 | 1361.93 | 8.85 | 0.01 |
| H | 4.5 | 0.90 | 0.88 | 0.02 | 0.01 | 0.13 | 1362.26 | 9.18 | 0.01 |
| LQHP | 3.5 | 0.91 | 0.89 | 0.02 | 0.01 | 0.12 | 1363.08 | 10.00 | 0.01 |
| LQH | 3.5 | 0.90 | 0.89 | 0.02 | 0.01 | 0.12 | 1363.91 | 10.83 | 0.00 |
| LQHP | 4 | 0.90 | 0.89 | 0.02 | 0.01 | 0.13 | 1364.12 | 11.04 | 0.00 |
| LQH | 3 | 0.90 | 0.89 | 0.02 | 0.01 | 0.10 | 1364.21 | 11.13 | 0.00 |
| H | 3.5 | 0.90 | 0.89 | 0.02 | 0.01 | 0.12 | 1364.38 | 11.30 | 0.00 |
| LQ | 0.5 | 0.91 | 0.89 | 0.03 | 0.04 | 0.12 | 1364.44 | 11.35 | 0.00 |
| LQHP | 2 | 0.91 | 0.90 | 0.02 | 0.01 | 0.15 | 1365.28 | 12.19 | 0.00 |
| H | 5 | 0.90 | 0.88 | 0.02 | 0.01 | 0.15 | 1365.58 | 12.50 | 0.00 |
| LQH | 5 | 0.90 | 0.89 | 0.02 | 0.03 | 0.12 | 1365.62 | 12.54 | 0.00 |
| LQ | 4.5 | 0.89 | 0.88 | 0.02 | 0.02 | 0.12 | 1365.89 | 12.80 | 0.00 |
| LQ | 1.5 | 0.90 | 0.88 | 0.02 | 0.00 | 0.14 | 1366.20 | 13.12 | 0.00 |
| LQ | 5 | 0.89 | 0.88 | 0.02 | 0.02 | 0.12 | 1366.55 | 13.47 | 0.00 |
| LQH | 4 | 0.90 | 0.89 | 0.02 | 0.01 | 0.13 | 1366.75 | 13.67 | 0.00 |
| LQ | 2.5 | 0.90 | 0.88 | 0.02 | 0.02 | 0.12 | 1366.80 | 13.72 | 0.00 |
| LQ | 1 | 0.91 | 0.89 | 0.02 | 0.04 | 0.12 | 1367.28 | 14.19 | 0.00 |
| LQ | 4 | 0.89 | 0.88 | 0.02 | 0.02 | 0.12 | 1367.52 | 14.44 | 0.00 |
| LQHP | 5 | 0.90 | 0.89 | 0.02 | 0.01 | 0.13 | 1367.78 | 14.70 | 0.00 |
| LQ | 3.5 | 0.89 | 0.88 | 0.02 | 0.02 | 0.12 | 1368.27 | 15.19 | 0.00 |
| LQ | 3 | 0.90 | 0.88 | 0.02 | 0.02 | 0.12 | 1368.97 | 15.89 | 0.00 |
| LQ | 2 | 0.90 | 0.88 | 0.02 | 0.02 | 0.14 | 1369.00 | 15.92 | 0.00 |
| LQH | 4.5 | 0.90 | 0.89 | 0.02 | 0.01 | 0.13 | 1369.53 | 16.45 | 0.00 |
| LQHP | 4.5 | 0.90 | 0.89 | 0.02 | 0.01 | 0.13 | 1370.86 | 17.78 | 0.00 |
| LQHP | 1.5 | 0.91 | 0.89 | 0.02 | 0.01 | 0.13 | 1373.47 | 20.39 | 0.00 |
| H | 1 | 0.92 | 0.90 | 0.03 | 0.01 | 0.12 | 1376.80 | 23.72 | 0.00 |
| LQH | 2.5 | 0.90 | 0.89 | 0.02 | 0.01 | 0.10 | 1377.90 | 24.81 | 0.00 |
| H | 1.5 | 0.91 | 0.90 | 0.02 | 0.01 | 0.13 | 1378.47 | 25.39 | 0.00 |
| LQH | 1.5 | 0.91 | 0.89 | 0.02 | 0.01 | 0.15 | 1378.73 | 25.65 | 0.00 |
| LQHP | 1 | 0.92 | 0.89 | 0.03 | 0.04 | 0.15 | 1379.17 | 26.09 | 0.00 |
| LQH | 2 | 0.91 | 0.89 | 0.02 | 0.01 | 0.12 | 1385.30 | 32.22 | 0.00 |
| L | 5 | 0.88 | 0.87 | 0.02 | 0.02 | 0.13 | 1385.78 | 32.70 | 0.00 |
| L | 4 | 0.88 | 0.87 | 0.02 | 0.02 | 0.12 | 1387.71 | 34.63 | 0.00 |
| L | 2 | 0.88 | 0.87 | 0.03 | 0.02 | 0.10 | 1389.47 | 36.39 | 0.00 |
| L | 2.5 | 0.88 | 0.87 | 0.02 | 0.02 | 0.10 | 1389.61 | 36.53 | 0.00 |
| L | 3 | 0.88 | 0.87 | 0.02 | 0.02 | 0.10 | 1389.78 | 36.70 | 0.00 |
| L | 3.5 | 0.88 | 0.87 | 0.02 | 0.02 | 0.10 | 1390.00 | 36.92 | 0.00 |
| L | 4.5 | 0.88 | 0.87 | 0.02 | 0.02 | 0.12 | 1390.56 | 37.48 | 0.00 |
| L | 1.5 | 0.88 | 0.87 | 0.03 | 0.02 | 0.10 | 1391.69 | 38.61 | 0.00 |
| LQH | 1 | 0.92 | 0.89 | 0.03 | 0.04 | 0.15 | 1395.53 | 42.45 | 0.00 |
| L | 1 | 0.88 | 0.86 | 0.03 | 0.02 | 0.10 | 1396.77 | 43.69 | 0.00 |
| L | 0.5 | 0.88 | 0.86 | 0.03 | 0.02 | 0.12 | 1403.60 | 50.52 | 0.00 |
| H | 0.5 | 0.93 | 0.89 | 0.04 | 0.05 | 0.17 | 1472.59 | 119.50 | 0.00 |
| LQH | 0.5 | 0.93 | 0.88 | 0.05 | 0.07 | 0.19 | 1538.29 | 185.21 | 0.00 |
| LQHP | 0.5 | 0.93 | 0.88 | 0.05 | 0.07 | 0.17 | 1552.60 | 199.52 | 0.00 |
