# Supplementary material for: Molecular systematics of the Reithrodontomys tenuirostris group (Rodentia: Cricetidae) highlighting the Reithrodontomys microdon species complex
Source: J Mammal. 2021 Dec 11;103(1):29–44. doi: 10.1093/jmammal/gyab133 (PMC8789765; doi:10.1093/jmammal/gyab133)
Supplement: gyab133_suppl_Supplementary_Data_2 [file gyab133_suppl_supplementary_data_2.docx]

Supplementary Data SD2


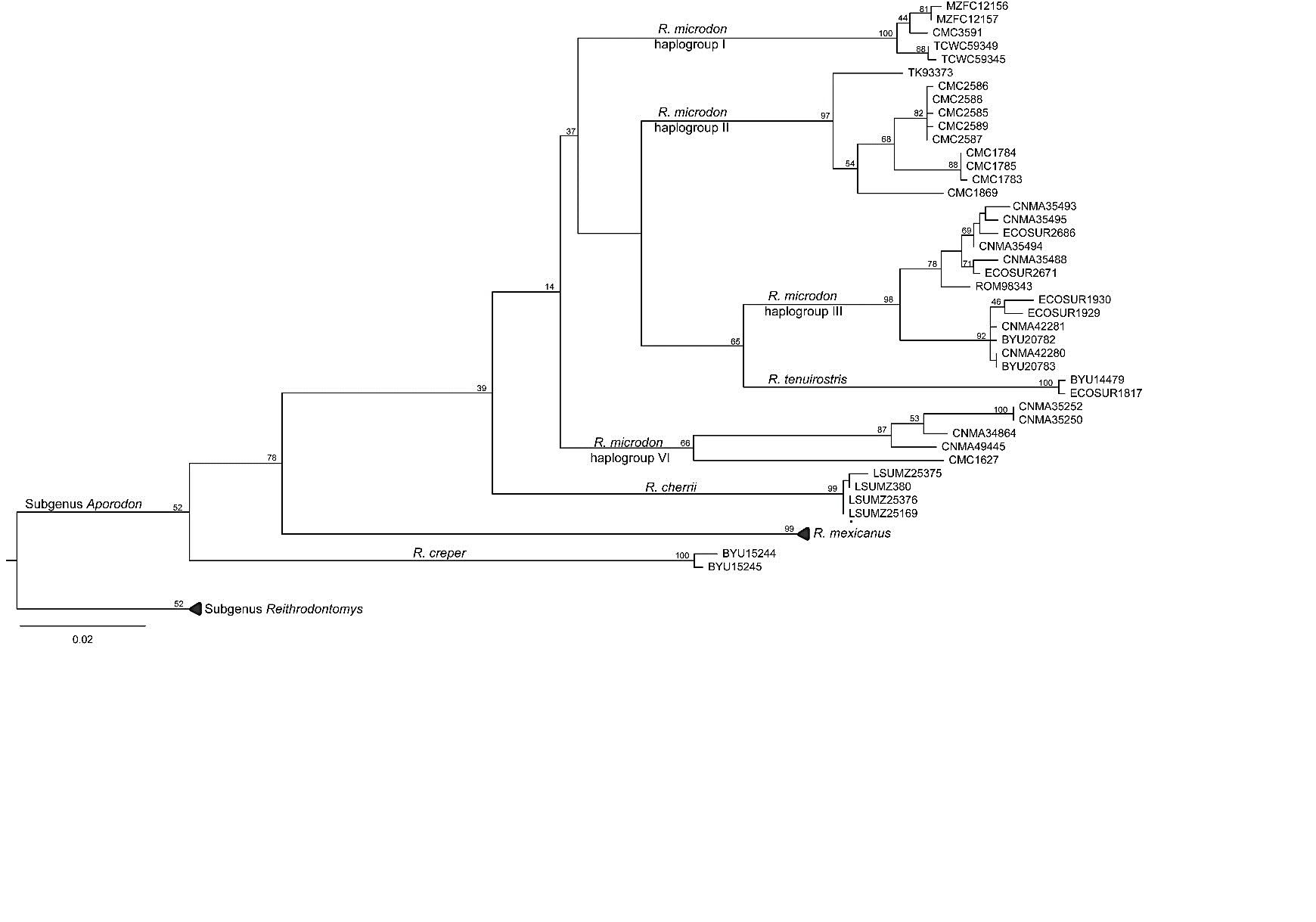


Phylogenetic relationships among species of the *Reithrodontomys tenuirostris* group (Rodentia: Cricetidae) using a concatenated sequences data set (Cytochrome *b* + Intron 7 of the beta fibrinogen) and the reconstructive method of Maximum Likelihood (ML). Values above branches represent nodal support for ML analysis. Terminal labels are named according to mammal collection voucher numbers (see Appendix I).
