# Supplementary material for: Molecular systematics of the Reithrodontomys tenuirostris group (Rodentia: Cricetidae) highlighting the Reithrodontomys microdon species complex
Source: J Mammal. 2021 Dec 11;103(1):29–44. doi: 10.1093/jmammal/gyab133 (PMC8789765; doi:10.1093/jmammal/gyab133)
Supplement: gyab133_suppl_Supplementary_Data_3 [file gyab133_suppl_supplementary_data_3.docx]

Supplementary Data SD3


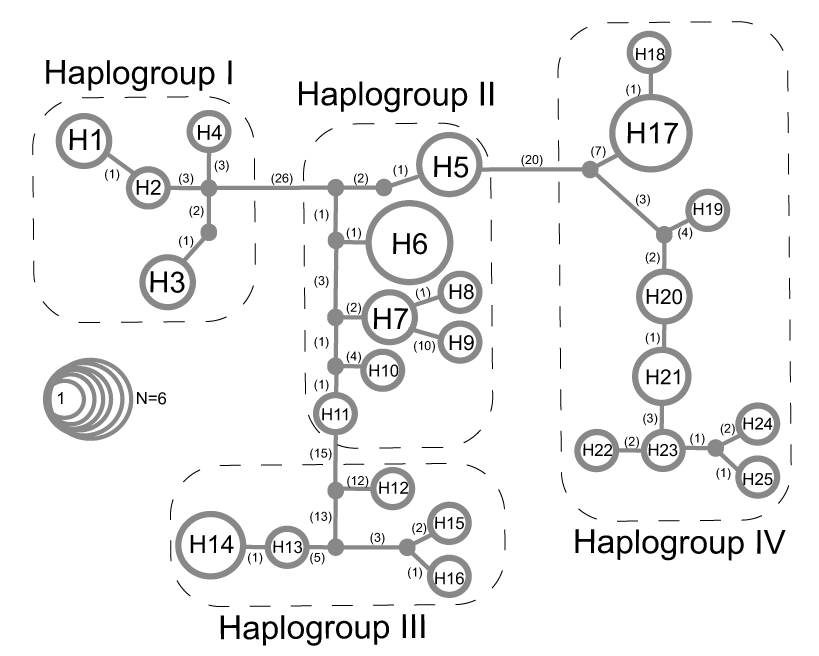


TCS network for Cytochrome *b* haplotypes of *Reithrodontomys microdon* (Rodentia: Cricetidae). Haplogroup II included *R. bakeri* haplotypes. Numbers in parentheses represent mutational steps between haplotypes, and the filled gray circles are theoretical consensus sequences (unsampled or extinct).
