# Supplementary material for: Molecular systematics of the Reithrodontomys tenuirostris group (Rodentia: Cricetidae) highlighting the Reithrodontomys microdon species complex
Source: J Mammal. 2021 Dec 11;103(1):29–44. doi: 10.1093/jmammal/gyab133 (PMC8789765; doi:10.1093/jmammal/gyab133)
Supplement: gyab133_suppl_Supplementary_Appendix_I [file gyab133_suppl_supplementary_appendix_i.docx]

**Appendix I**

List of *Reithrodontomys* specimens with collecting localities and GenBank accessions numbers for Cytochrome *b* and Intron 7 of the beta fibrinogen genes (asterisks depict DNA sequences generated in this study). Abbreviations for countries and mammal collections (Dunnam et al. 2018^+^) housing the specimens we included are as follows: CR = Costa Rica; GU = Guatemala; MX = Mexico; USA = United States of America; ASNHC = Angelo State Natural History Collections; BYU = Brigham Young University; CMC = Colección de Mamíferos del CIByC; CNMA = Colección Nacional de Mamíferos, Universidad Nacional Autónoma de México; ECO-SC-M = Colección de Mamíferos de El Colegio de la Frontera Sur, San Cristóbal; LSUMNS = Louisiana State University Museum of Zoology; MVZ = Museum of Vertebrate Zoology; MZFC = Colección de Mamíferos, Museo de Zoología "Alfonso L. Herrera", Universidad Nacional Autónoma de México; ROM = Royal Ontario Museum; TCWC = The Biodiversity Research and Teaching Collections, Texas A&M University; TK = Mammal Collection, Texas Tech University.

| Current species name | N | Locality | Museum/Mammal Collection Voucher | GenBank Accession (*Cytb*) | GenBank Accession (*Fgb*) |
| --- | --- | --- | --- | --- | --- |
| *R. microdon* | 1 | MX-Tamaulipas, Estación Biología Los Cedros Gómez Farias, Reserva de la Biosfera El Cielo, 320 m | TCWC59345 | MW117039* | MW117073* |
| *R. microdon* | 1 | MX-Tamaulipas, Hotel El Pino, Alta Cima, Reserva de la Biosfera El Cielo, 910 m | TCWC59347 | MW117040* |  |
| *R. microdon* | 1 | MX-Tamaulipas, San José, Reserva de la Biosfera El Cielo, 1320 m | TCWC59349 | MW117041* | MW117072* |
| *R. microdon* | 1 | MX-Veracruz, Sierra de Otontepec 7.6 km SE Citlaltepec, 1100 m | CMC3591 | MW367885* | MW367886* |
| *R. microdon* | 2 | MX-San Luis Potosí, Las Guapas, Rayón, 1062 m | MZFC12156 MZFC12157 | MW117042*  MW117043* | MW117070*  MW117071* |
| *R. microdon* | 1 | MX-Michoacán, Municipio Hidalgo, Mil Cumbres 8.9 km (by road), 2625 m | CMC1869 | MW117053* | MW117089* |
| *R. microdon* | 3 | MX-Estado de México, municipio Zacualpan, 8 km SW Zacualpan by road Zacualpan-Mamatla, 2315 m | CMC1783  CMC1784  CMC1785 | MW117056*  MW117054*  MW117055* | MW117086*  MW117087*  MW117092* |
| *R. microdon* | 1 | MX-Estado de México, 10.5 km SE de Amecameca by road Amecameca-Tlamacas, 2970 m | CMC197 | MW117057* |  |
| *R. microdon* | 5 | MX-Morelos, Municipio Tepoztlán, 6.5 km E of Coajumulco, old railroad track, 2500 m | CMC2585  CMC2586  CMC2587  CMC2588  CMC2589 | MW117062*  MW117058*  MW117060*  MW117061*  MW117059* | MW117091*  MW117084*  MW117090*  MW117085*  MW117078* |
| *R. microdon* | 1 | MX-Guerrero, Municipio Malinaltepec, 3 km E of El Tejocote, 2620 m | CMC1627 | MW117063* | MW117080* |
| *R. microdon* | 1 | MX-Oaxaca, Municipio San Juan Lachao, El Polvorín 5.3 km S (by road) from turn off to Lachao Viejo, 1735 m | CNMA42282 | AY859461 |  |
| *R. microdon* | 3 | MX-Oaxaca, Municipio Santa María Tlahuitoltepec, Santa María Yacochi 4.5 km N Cerro Zempoaltepelt, 2450 m | CNMA35248 CNMA35249 CNMA35250 | MW117064*  MW117065*  MW117066* | MW117082* |
| *R. microdon* | 1 | MX-Oaxaca, Municipio Santa María Tlahuitoltepec, Santa María Yacochi 5 km N Cerro Zempoaltepelt, 2450 m | CNMA34864 | MW117068* | MW117081* |
| *R. microdon* | 1 | MX-Oaxaca, Municipio Santa María Tlahuitoltepec, Santa María Yacochi Cerro Zempoaltepec, 2450 m | CNMA35252 | AY293819 | MW117083* |
| *R. microdon* | 1 | MX-Oaxaca, Ixtepeji, 1892 m | CNMA49445 | MW117067* | MW117079* |
| *R. microdon* | 2 | MX-Chiapas, Municipio El Porvenir, Cerro Mozotal 30 km N Motozintla by road Buenos Aires- El Porvenir | ECO-SC-M1929  ECO-SC-M1930 | MW117047*  MW117046* | MW117103*  MW117100* |
| *R. microdon* | 4 | MX-Chiapas, Municipio El Porvenir, Cerro Mozotal, 2930 m | BYU20782 BYU20783  CNMA42280 CNMA42281 | MW117049*  MW117048*  AY859460 AY859459 | MW117105*  MW117111*  MW117104*  MW117110* |
| *R. microdon* | 5 | MX-Chiapas, Municipio Chamula, Cerro Tzontehuitz 13 km NE San Cristóbal de las Casas, 2880 m | CNMA35488 CNMA35493 CNMA35494 CNMA35495 BYU14476 | AY859454 AY859455 AY859456 AY859457 AY293818 | MW117112*  MW117107*  MW117108*  MW117109* |
| *R. microdon* | 2 | MX-Chiapas, Reserva Ecológica Huitepec Hillside W, 2 km NE San Cristóbal de las Casas | ECO-SC-M2686  ECO-SC-M2671 | MW117052*  MW117051* | MW117102*  MW117101* |
| *R. microdon* | 2 | GU-Huehuetenango, 16 km NW of Santa Eulalia (by road) | ROM98382  ROM98343 | EF990014  MW117050* | MW117106* |
| *R. microdon* | 1 | GU-Huehuetenango, 12 km NW of Santa Eulalia (by road) | ROM98300 | AY859458 |  |
| *R. bakeri* | 2 | MX-Guerrero, Municipio Chilpancingo, Omiltemi | CNMA40380  CNMA40381 | AY293815  AY293816 |  |
| *R. bakeri* | 3 | MX-Guerrero, Municipio Chichihualco, Filo de Caballo | TK93372  TK93373  TK93374 | AY293812  AY293813  AY293814 | MW117088* |
| *R. tenuirostris* | 1 | MX-Chiapas, Municipio El Porvenir, Cerro Mozotal, 2930 m | CMC736 | MW117044* |  |
| *R. tenuirostris* | 1 | MX-Chiapas, Municipio Chamula, Cerro Tzontehuitz 13km NE San Cristóbal de las Casas, 2880 m | BYU14479 | AY859463 | MW117095* |
| *R. tenuirostris* | 1 | MX-Chiapas, Municipio Chamula, Cerro Tzontehuitz 11 km NE San Cristóbal de Las Casas, 2890 m | ECO-SC-M1817 | MW117045* | MW117096* |
| *R. creper* | 1 | CR-Heredia, Municipio Heredia, Los Ángeles de Paso Llano, Barva, San José de la Montaña, 2050 m | BYU15243 |  | MW117099* |
| *R. creper* | 2 | CR-Heredia, Municipio Heredia, San José de la Montaña, 2050 m | BYU15244  BYU15245 | AY859429  AY859430 | MW117097*  MW117098* |
| *R. creper* | 2 | CR-Cartago, 12 km N of Potrero Cerrado (by road), Rio Birrís | ROM97320  ROM97321 | AY859428  DQ861372 |  |
| *R. creper* | 1 | CR-Cartago, 12 km N of Porter, Rio Birrís | ROM97311 | EF989996 |  |
| *R. creper* | 2 | CR-Cartago, Parque Nacional Volcán Irazú | ROM116798  ROM116799 | EF989999  EF989998 |  |
| *R. cherrii* | 3 | CR-San José, 1 km (by road) SW Poás, 1500 m | LSUMNS25169  LSUMNS25375  LSUMNS25376 | MW117038*  MW117036*  AY293821 | MW117074*  MW117076*  MW117075* |
| *R. cherrii* | 1 | CR-Cartago | LSUMNS380 | MW117037* | MW117077* |
| *R. mexicanus* | 1 | MX-Oaxaca, Municipio Santiago Comaltepec, 11 km SW (by road) La Esperanza | BYU15426 | AY859449 | MW117093* |
| *R. mexicanus* | 1 | GU- Baja Verapaz, 5 km E of Puruhla | ROM98467 | AY859451 | MW117094* |
| *R. brevirostris* | 1 | CR-Cartago, Colima Tapanti 1.6 km S Tapanti Bridge over Rio Grande de Orosi, 1290 m | MVZ174401 | AF108709 |  |
| *R. brevirostris* | 1 | CR-Alajuela, Parque Nacional Juan Castro Blanco, 10 km E of Sucre | ROM116804 | EF990017 |  |
| *R. gracilis* | 2 | MX-Yucatán, Laguna Becanchen | ROMFN30426  ASNHC6370 | AY293817  AY859431 |  |
| *R. megalotis* | 1 | USA-California, Berkeley, Contra Costa Co., 300-400 m Big Springs picnic area on Arroyo trail, Tilden Regional Park, 392 m | MVZ206953 | KR611945 |  |
| *R. megalotis* | 1 | USA-Texas, Lubbock Co., Lubbock Lake Landmark State Historical Park, 975 m | TK22460 |  | KJ697789 |
| *R. megalotis* | 1 | MX-Puebla, Municipio Teziutlán, 4.7 km NE Teziutlán (by road), 1750 m | CMC1072 | HQ269732 | HQ269795 |
| *R. sumichrasti* | 2 | MX-Chiapas, Municipio El Porvenir, 2930 m | CMC680  BYU20784 | MW117069*  HQ269707 | MW117113*  HQ269781 |
| *R. fulvescens* | 1 | MX-Oaxaca, Municipio Pinotepa Nacional, Rio de la Arena, 6 km E (by road) Pinotepa Nacional, 60 m | BYU20914 | HQ269730 | HQ269794 |
| *R. fulvescens* | 1 | MX-Chiapas, Municipio Mazapa de Madero, 5 km NE (by road) Mazapa de Madero, 1100 m | CNMA42278 | AY859465 |  |
| *R. fulvescens* | 1 | USA-Oklahoma, Mcintosh Co., 1.9 km E Dustin | TK23469 |  | AY274211 |

^+^Dunnum, J. L., B. S. McLean, and R. C. Dowler. 2018. Mammal collections of the Western Hemisphere: a survey and directory of collections. Journal of Mammalogy 99:1307–1322.
